# Acute cholangitis in intensive care units: clinical, biological, microbiological spectrum and risk factors for mortality: a multicenter study

**DOI:** 10.1186/s13054-021-03480-1

**Published:** 2021-02-06

**Authors:** Jean-Rémi Lavillegrand, Emmanuelle Mercier-Des-Rochettes, Elodie Baron, Frédéric Pène, Damien Contou, Raphael Favory, Sébastien Préau, Arnaud Galbois, Chloé Molliere, Arnaud-Félix Miailhe, Jean Reignier, Mehran Monchi, Claire Pichereau, Sara Thietart, Thibault Vieille, Gael Piton, Gabriel Preda, Idriss Abdallah, Marine Camus, Eric Maury, Bertrand Guidet, Guillaume Dumas, Hafid Ait-Oufella

**Affiliations:** 1 Assistance Publique – Hôpitaux de Paris (AP-HP), Service de médecine intensive et réanimation, Hôpital Saint-Antoine, 184 rue du Faubourg Saint-Antoine, 75571 Paris Cedex 12, France; 2 Sorbonne Université, Paris, France; 3 Groupe Hospitalier Sud Île-De-France (GHSIF), Service de réanimation polyvalente, Hôpital de Melun-Sénart, 77000 Melun, France; 4 Assistance Publique – Hôpitaux de Paris (AP-HP), Service de médecine intensive et réanimation, Hôpital Cochin, 75014 Paris Cedex 12, France; 5 Centre Hospitalier Argenteuil, Service de réanimation polyvalente et unité de surveillance continue, 95107 Argenteuil, France; 6 Centre Hospitalier Universitaire Lille, Service de réanimation générale, Hôpital Salengro, 59037 Lille, France; 7 Service de réanimation polyvalente, Hôpital Privé Claude Galien, 91480 Quincy-sous-Sénart, France; 8 Service de médecine intensive et réanimation, Centre Hospitalier Universitaire Nantes, Hôtel-Dieu, 44000 Nantes, France; 9 Centre Hospitalier Intercommunal Poissy Saint-Germain-en-Laye, Service de réanimation, Hôpital de Poissy, 78303 Poissy, France; 10 Centre Hospitalier Régional Universitaire Besançon, Service de réanimation médicale, Hôpital Jean Minjoz, 25030 Besançon, France; 11 Centre Hospitalier de Saint-Denis, Service de réanimation et soins continus, Hôpital Delafontaine, 93205 Saint-Denis, France; 12 Centre Hospitalier Sud Seine-et-Marne, Service de réanimation, Hôpital Fontainebleau, 77300 Fontainebleau, France; 13 Inserm U970, Centre de Recherche Cardiovasculaire de Paris (PARCC), Paris, France; 14 Assistance Publique – Hôpitaux de Paris (AP-HP), Centre d’endoscopie digestive, Hôpital Saint-Antoine, Paris, France

**Keywords:** Cholangitis, Outcome, Prognosis, Microbiology, Intensive care unit

## Abstract

**Background:**

Little is known on the outcome and risk factors for mortality of patients admitted in Intensive Care units (ICUs) for Acute cholangitis (AC).

**Methods:**

Retrospective multicenter study included adults admitted in eleven intensive care units for a proven AC from 2005 to 2018. Risk factors for in-hospital mortality were identified using multivariate analysis.

**Results:**

Overall, 382 patients were included, in-hospital mortality was 29%. SOFA score at admission was 8 [5–11]. Biliary obstruction was mainly related to gallstone (53%) and cancer (22%). Median total bilirubin and PCT were respectively 83 µmol/L [50–147] and 19.1 µg/L [5.3–54.8]. Sixty-three percent of patients (*n*  = 252) had positive blood culture, mainly Gram-negative bacilli (86%) and 14% produced extended spectrum beta lactamase bacteria. At ICU admission, persisting obstruction was frequent (79%) and biliary decompression was performed using therapeutic endoscopic retrograde cholangiopancreatography (76%) and percutaneous transhepatic biliary drainage (21%). Adjusted mortality significantly decreased overtime, adjusted OR for mortality per year was 0.72 [0.54–0.96] (*p* = 0.02). In a multivariate analysis, factors at admission associated with in-hospital mortality were: SOFA score (OR 1.14 [95% CI 1.05–1.24] by point, *p* = 0.001), lactate (OR 1.21 [95% CI 1.08–1.36], by 1 mmol/L, *p* < 0.001), total serum bilirubin (OR 1.26 [95% CI 1.12–1.41], by 50 μmol/L, *p* < 0.001), obstruction non-related to gallstones (*p* < 0.05) and AC complications (OR 2.74 [95% CI 1.45–5.17], *p* = 0.002). Time between ICU admission and biliary decompression > 48 h was associated with in-hospital mortality (adjusted OR 2.73 [95% CI 1.30–6.22], *p* = 0.02).

**Conclusions:**

In this large retrospective multicenter study, we found that AC-associated mortality significantly decreased overtime. Severity of organ failure, cause of obstruction and local complications of AC are risk factors for mortality, as well as delayed biliary drainage > 48 h.

## Introduction

Acute cholangitis (AC), also called ascending cholangitis, is a bacterial infection of the biliary system, due to partial or complete obstruction of the bile duct or hepatic ducts. The most common cause of biliary obstruction is gallstone or malignant stricture, but obstruction caused by primary sclerosing cholangitis, amyloid depositions or parasite biliary invasions can also be observed. Classically, patients with AC present with persisting high fever for more than 24 h, right upper quadrant abdominal pain and jaundice. The International Consensus Meeting held in Tokyo in 2006 proposed to use inflammatory response biomarkers, liver function tests and imaging in order to optimize diagnosis, localize biliary obstruction and guide the etiological diagnosis [1].

AC diagnosis and management should be considered as an emergency because it may be responsible for life-threatening organ failure and death. Intensive Care unit (ICU) admission of patients with AC is not rare, as it is responsible for 5% of septic shocks [2] and for 10 to 29% of intra-abdominal sepsis [3–5]. The cornerstone of the management of AC is based on fluid resuscitation, antibiotics, organ support therapy, and biliary drainage. However, not all ICUs have access to endoscopic retrograde cholangiopancreatography (ERCP), interventional radiology or hepatobiliary surgery at night or on week-end.

Our study aimed to describe over a 14-year period the demographical, clinical and microbial patterns of critically ill patients admitted in ICUs for AC, to describe therapeutic management and analyze risk factors for in-hospital mortality, including time to biliary decompression.

## Material and methods

### Study design

Between January 2005 and March 2018, all adult patients (≥ 18 years of age) admitted in 11 French ICUs (cf. Additional files 1 and 2) for acute cholangitis were identified by searching hospital databases for codes K803 or K830 as a principal or associated diagnosis according to the International Classification of Diseases (10^th^ version). Acute cholangitis was defined by the association of fever, jaundice, imaging abnormalities and/or biliary obstruction, according to the International Guidelines [6, 7].

### Data collection

Data were collected retrospectively by local investigators using electronic case report forms (eCRF) then centralized and anonymized. The following data were collected: age, gender, cardiovascular diseases (coronary artery diseases or chronic heart failure), hypertension, other comorbidities (e.g. diabetes mellitus, cancer, immune deficiency, cirrhosis, chronic renal failure), clinical symptoms (abdominal pain, jaundice, diarrhea, chills) at ICU admission. Disease severity was evaluated using the Simplified Acute Physiology Score (SAPS II), the Sequential Organ Failure Assessment (SOFA) score, and the use of organ support therapy.

Specific AC-related parameters occurring during ICU stay were also recorded: presence of liver abscesses; parameters related to biliary drainage (method, time from admission), ERCP complications, microbial analysis, anti-microbial therapy. Non-optimal biliary drainage was defined as persistent partial biliary obstruction after the first decompression procedure. Sepsis and septic shock were defined using the Third International Consensus Definitions for Sepsis and Septic Shock (Sepsis-3) [8]. Anti-microbial therapy was considered as appropriate if the first line therapy was active on the isolated pathogen (blood and/or biliary cultures) according to the EUCAST guidelines [9].

All authors had access to the study data and reviewed and approved the final manuscript.

Quality was assessed according to the STROBE checklist.

### Statistical analysis

Continuous variables were described as median [interquartile ranges (IQRs)] and categorical variables as proportions. Comparisons were made using the Fisher’s exact test or Wilcoxon rank-sum test when appropriate. First, factors associated with in-hospital mortality (model 1) were assessed using a multivariate mixed logistic model, considering the center as a random variable. Covariates associated with outcome at the 0.2 level in univariate analysis were selected upon clinical relevance and were included in the model as fixed effect. A multiple backward stepwise selection procedure eliminated those variables with an exit threshold set at *p* = 0.05, after testing for collinearity between variables and checking the assumption of log-linearity as described above.

Second, to investigate the prognostic significance of time to biliary drainage on in-hospital mortality (model 2), the analysis took death into account as a competing risk of drainage. Thus, we used a Landmark analysis restrained to patients still alive 3 days after ICU admission. This landmark time was fixed before the analysis, because it was considered as the maximum time to obtain an available technical center. Then the time to decompression was assessed with multivariate mixed model with center as a random variable and adjusted on SOFA score (day 3), obstacle nature, local complications of cholangitis, prothrombin time and appropriate empirical antibiotics.

To handle missing values as potential confounders, multiple imputation with chained equation was used. Primary analyses were performed on the complete cases, assuming missing completely at random covariates. Then, sensitivity analyses for such assumptions were performed, based on multiple imputation with chained equation.

To test for the significance of the center effects, we used permutations test, a recommended approach to test for random effect (for details [10]).

Odd-Ratios (OR) in the two final models are given with their 95% confidence intervals (CI). All tests were two-sided and p-values lower than 5% were considered to indicate significant associations. Analyses were performed using R statistical platform, version 3.0.2 (https://cran.r-project.org/).

## Results

### General characteristics

Over a 14-year period, 382 patients were included in 11 ICUs (5 tertiary hospitals, 5 general hospitals and 1 private hospital) in France (Additional files 1 and 2). Median inclusion number per center was 28 [18–48]. The median age was 72 [62–81] years with a twofold higher incidence of men (63%). Patient characteristics are summarized in Table 1. Comorbidities were frequent, notably diabetes (*n* = 112, 30%), cardiovascular diseases (*n* = 301, 79%) and active solid tumors (*n* = 118, 31%). Abdominal pain (73%) and jaundice (63%), which are classical clinical signs of AC, were frequently observed but fever (defined by a temperature > 38 °C) was only reported in 116 patients (31%). The most commonly used imaging method for diagnosis of AC was abdominal CT-scan (167/382; 46%), and less frequently ultrasonography (65/382; 18%). As expected, the 2 main causes of biliary obstruction were gallstones (201/382 patients; 53%) and tumors (85/382 patients; 22%). AC due to primary sclerosing cholangitis was rare (15/382, 4%).

**Table 1:** General characteristics of the 382 patients included

Parameters	*n* (%) or Med. [IQR]
Age	72 [62–81]
BMI	26 [23–81]
Male gender	242 (63)
SAPS2	50 [38–64]
SOFA at admission	8 [5–11]
*Comorbidities*	
Diabetes	116 (30)
Hypertension	235 (62)
Cardiovascular disease	301 (79)
Chronic kidney disease	46 (12)
Cirrhosis	20 (5)
Cancer and hematologic malignancies	141 (37)
*Organ support therapy during ICU stay*	
Invasive mechanical ventilation	144 (38)
Invasive mechanical ventilation duration: days	5 [2–10]
Renal replacement therapy	91 (26)
Vasopressor infusion	222 (61)
Vasopressor infusion duration: hours	48 [24–91]
Persisting obstruction at ICU admission	298 (79)
*Biliary drainage procedure*	
Percutaneous derivation	78 (21)
ERCP	285 (76)
Surgery	28 (7)
Complications before ERCP	
Associated pancreatitis	94 (25)
Liver abscess	31 (8)
Complications after ERCP	
Bleeding	22 (9)
Gastrointestinal perforation	7 (3)
Pancreatitis	10 (4)
Death in ICU	80 (21)
Death in hospital	105 (29)
Length of stay in ICU: days	5 [3–10]
Length of stay in hospital: days	6 [1–11]

SAPS2, simplified acute physiology score; SOFA, Sepsis-related organ failure; ERCP, endoscopic retrograde cholangiopancreatography

At ICU admission, patients with AC had severe diseases, with sepsis (370/382; 97%), septic shock (170/382; 44%) and a high SOFA score (8 [5–11]) [8]. They needed frequent organ support therapies, including mechanical ventilation (38%), vasopressor infusion (61%) and renal-replacement therapy (26%) (Table 1). A high proportion of patients had renal failure, coagulopathy and lactic acidosis (Table 2). Finally, in-ICU mortality reached 21%, half of the deaths occurring within the first 10 days of ICU admission. Time between ICU admission and appropriate antibiotic therapy administration was 0 [− 4; 3] hours.

**Table 2:** Clinical and biological parameters at ICU admission

Parameters	*n* (%) or Med. [IQR]
*Clinical signs*	
Abdominal pain	278 (73)
Jaundice	240 (63)
Chills	118 (31)
Temperature	37.4 [36.5–38.3]
Temperature > 38.0 °C	116 (31)
*Biological parameters*	
Leucocytes (Giga/L)	15.29 [10.42–21.62]
Hemoglobin (g/dL)	11.1 [9.6–12.9]
Platelets (Giga/L)	160.0 [98.2–240.0]
Blood alanine aminotransferase (X ULN)	4.1 [2–8.2]
Blood aspartate aminotransferase (X ULN)	3.5 [1.6–7.2]
Total blood bilirubin (µmol/L)	83 [50–147]
Lipase (IU/L)	55 [18–276]
Serum creatinine (µmol/L)	143.5 [81–232]
Prothrombin time < 50%	125 (34)
APTT	1.2 [1–1.45]
Factor V < 50%	30 (11)
C-reactive protein (mg/L)	144.5 [75.25–233]
Procalcitonin (ng/mL)	19.11 [5.27–54.81]
Arterial lactate (mmol/L)	2.61 [1.5–4.6]

APTT, activated partial thromboplastin time; X ULN, X times upper limit normal

### Biliary drainage

Overall, biliary drainage was performed in 86% (*n* = 330/382) of the patients either before ICU admission (*n* = 32/330) or during ICU stay (*n* = 298/330). The median time to biliary drainage was 24 [9–48] hours. Endoscopic Retrograde Cholangiopancreatography (ERCP) was the first-line technique used for biliary drainage in 285 patients (*N* = 24 before ICU admission), associated with stent placement in 48% of the interventions (*n* = 137/285). Completion rate of ERCP within 24 or 48 h did not change during the 2005–2018 period. The procedure was described as optimal in 75% of the cases by the physician who performed the drainage. Non-optimal procedure was more frequently reported in non-gallstone related AC, when compared with gallstone related AC (11/38 *versus* 15/201; *p* = 0.006). The median time between ICU admission and biliary drainage was 39.5 [17.3–84.8] hours. Percutaneous biliary derivation was performed in 78 patients (21%) and surgical decompression was rarely done (*n* = 28, 7%).

Following ERCP procedure, 40 complications (14%) were reported including bleeding requiring red blood cell transfusion (*n* = 22, 9%); pancreatitis (*n* = 10, 4%) and gastro-intestinal perforation (*n* = 7, 3%).

### Microbiologic analysis

**Fig. 1:**
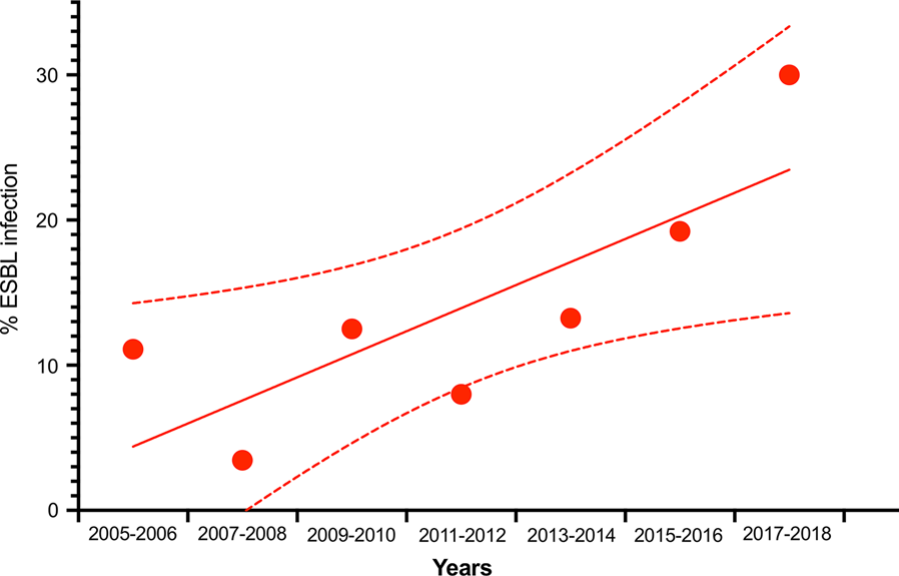
Observed annual percentage of Extended spectrum beta lactamase producing bacterial infections on documented acute cholangitis in ICUs. The line is a linear regression with 95% CIs

Positive blood culture was frequently reported in patients with AC (*n* = 252/373, 68%). Bile culture was only performed in 189/375 patients and was positive in 79% of the cases (149/189). When blood and bile were simultaneously collected (*n* = 189), both culture samples were positive in 56% of the cases (*n* = 106/189). Microbiological data are summarized in Table 3. In blood or bile culture, the most frequently identified bacteria were Gram-negative bacilli (68.5%) and Gram-positive (24.5%). Anaerobes (4.5%) and fungi (2.5%) were relatively uncommon. The most frequently identified bacteria were *Escherichia coli* (38%), *Klebsiella pneumoniae* (9%), *Enterococcus faecalis* (8%), *Enterococcus faecium* (7.5%), *Enterobacter cloacae* (6%) and *Pseudomonas aeruginosa* (5%)*.* Finally, an infection caused by Extended spectrum beta lactamase (ESBL)-producing bacteria was observed in 44 AC patients (12%). Interestingly, in our cohort the prevalence of ESBL-producing bacteria increases over time (*p* = 0.011, Chi-squared for a trend) (Fig. 1). In multivariate analysis, non-gallstone biliary tract obstacle was significantly associated with ESBL bacterial infection (OR 2.13 [1.11–4.21], *p* = 0.02) (Additional file 3).

**Table 3:** Microbiological data in blood and bile cultures

Germs	Blood	Bile
Samples performed	373	189
Positive culture	252 (68)	149 (79)
> 1 pathogen/culture	91 (36)	75 (40)
Total pathogens	379	261
Gram-negative bacilli	282 (74)	155 (59)
*Escherichia coli*	162 (43)	79 (30)
*Klebsiella pneumoniae*	39 (10)	18 (7)
*Enterobacter cloacae*	23 (6)	14 (5.5)
*Klebsiella oxytoca*	18 (5)	7 (2)
*Pseudomonas aeruginosa*	16 (4)	15 (6)
*Citrobacter freundii*	5 (1)	5 (2)
Others GNB	19 (5)	17 (6.5)
ESBL	38 (10)	27 (10)
Gram-positive bacilli	76 (20.5)	81 (31)
*Enterococcus faecium*	26 (7)	22 (8.5)
*Streptococcus spp.*	19 (5)	5 (2)
*Enterococcus faecalis*	17 (4.5)	34 (13)
*Staphylococcus aureus*	3 (1)	4 (1.5)
Others GP	11 (3)	16 (6)
Anaerobes	19 (5)	9 (3.5)
*Clostridium perfringens*	9 (2.5)	
*Bacteroides fragilis*	6 (1.5)	
*Clostridium difficile*	2 (0.5)	
Others	2 (0.5)	
Fungi	2 (0.5)	14 (5.5)
Cytomegalovirus		1 (0.5)
Cryptosporidium		1 (0.5)

GNB, Gram negative bacteria; ESBL, extended spectrum beta lactamase, GP, Gram positive

### Predictive factors of mortality

**Fig. 2:**
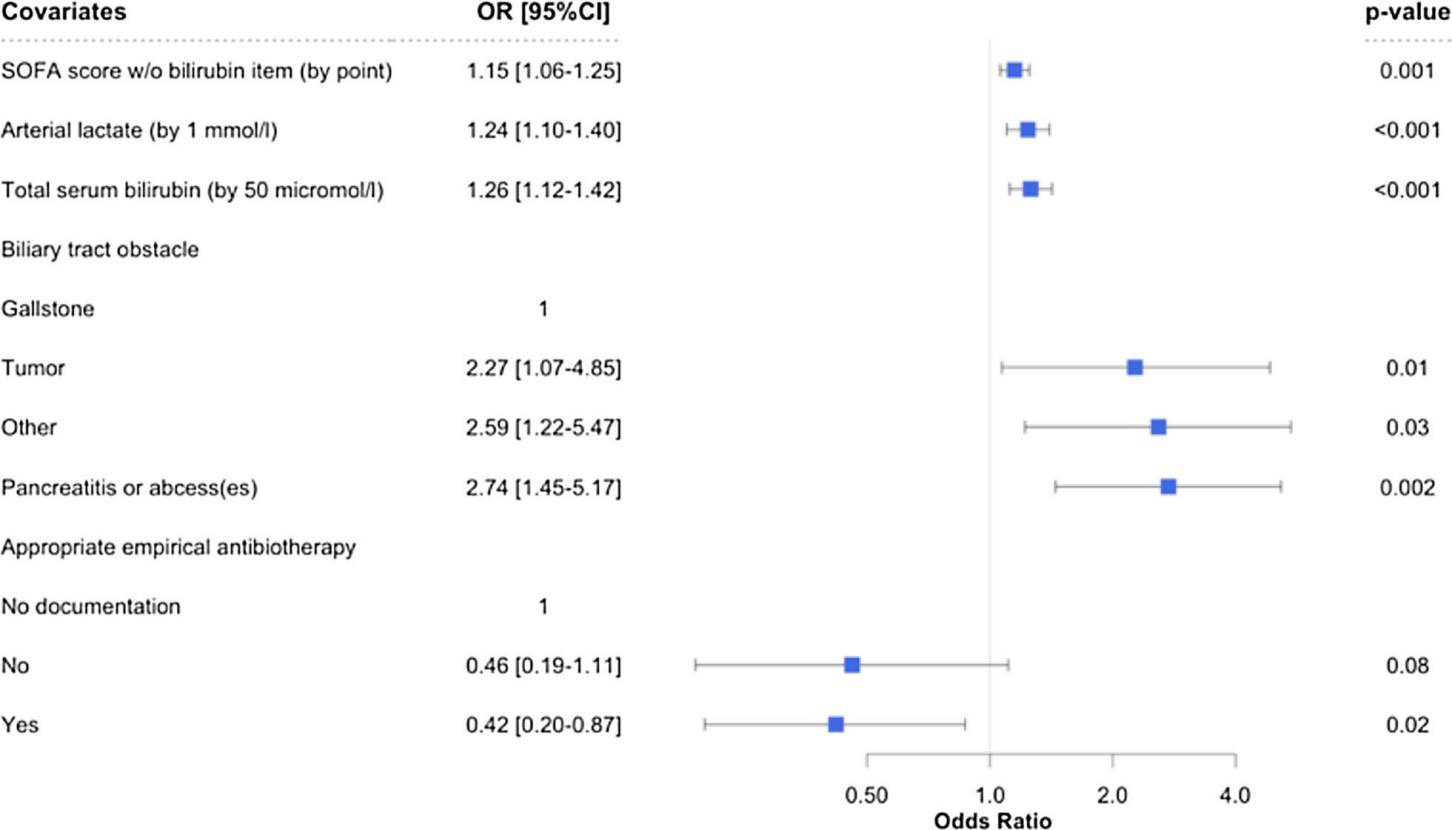
Factors associated with in-hospital mortality (multivariate analysis)

**Fig. 3:**
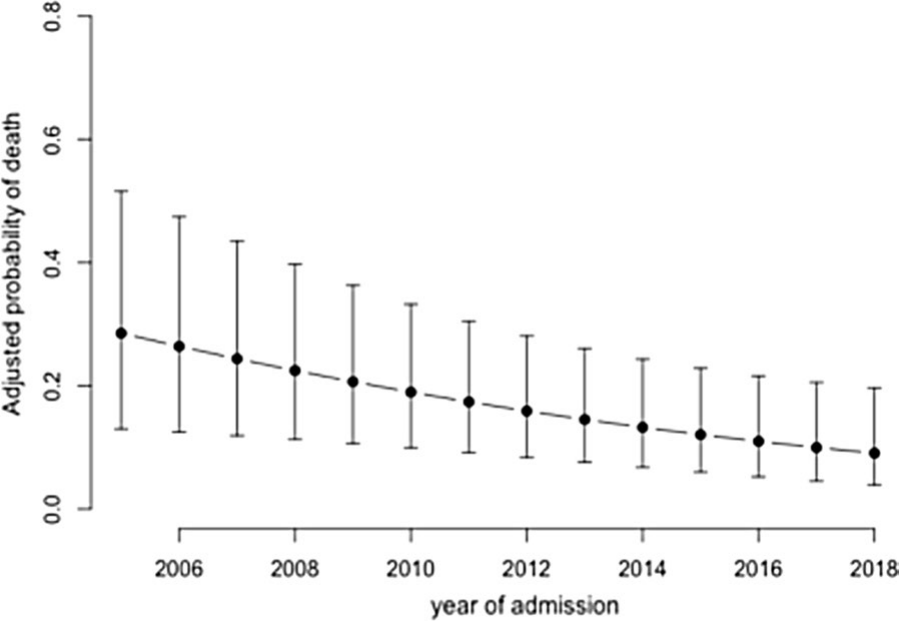
Adjusted probability of in-hospital mortality across years

Overall in-hospital mortality was 29%. Difference in AC-associated mortality between participating centers could not be ruled out (p-value for permutation test = 0.06) (Additional file 4). Several factors were significantly associated with in-hospital death (Fig. 2): SOFA score without bilirubin item, by point (OR 1.15 [95% CI 1.06–1.25], *p* = 0.001), arterial lactate, by 1 mmol/L (OR 1.24 [95% CI 1.10–1.40], *p* < 0.001), total serum bilirubin; by 50 µmol/L (OR 1.26 [95% CI 1.12; 1.42], *p* < 0.001), tumor as an etiology of AC (OR 2.27 [95% CI 1.07–4.85], *p* = 0.01), an initial AC-associated complication (OR 2.74 [95% CI 1.45–5.17], *p* = 0.002) and an appropriate empirical antibiotic therapy (OR 0.42 [95% CI 0.20–0.87], *p* = 0.02). In-hospital mortality significantly decreased overtime, adjusted OR for mortality per year was 0.72 [0.54–0.96] (*p* = 0.02) (Fig. 3).

**Fig. 4:**
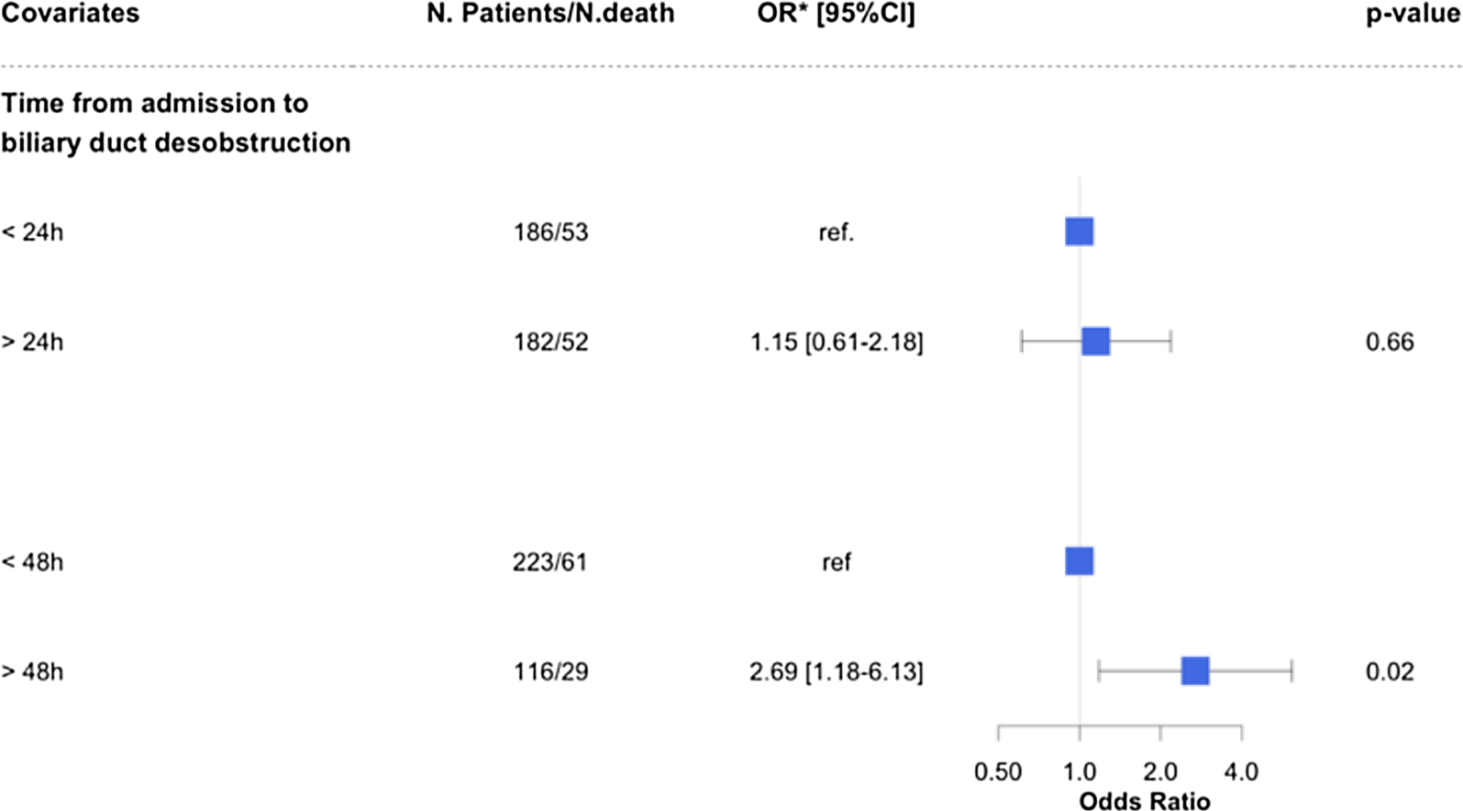
Adjusted effects of time from admission to biliary drainage on hospital mortality, SOFA score at day 0, cause of biliary obstacle, locoregional complications, PT < 50% and appropriate empirical antibiotic therapy

To analyze the effect of time from admission to biliary drainage on hospital mortality, we performed a landmark analysis adjusted on these factors with 2 predefined time points: 24 and 48 h after admission (Fig. 4). We found a significant relationship between delayed drainage (> 48 h) and hospital mortality (OR 2.69 [1.18–6.13], *p* = 0.02). However, no relationship between early drainage (< 24 h) and hospital mortality (OR 1.15 [0.61–2.18], *p* = 0.66) was found.

## Discussion

Here we describe the characteristics of 382 AC patients admitted in 11 ICUs in France over a 14-year period. In a multivariate regression model, we identified several independent predictive factors of mortality including SOFA score at admission; total bilirubin level, lactate level, non-gallstone-related biliary obstacles and local AC-related complications. Appropriate empirical antimicrobial therapy was associated with better survival whereas delayed biliary decompression (> 48 h) worsened patient outcome.

The most appropriate time to perform biliary drainage still remains unknown and has to be investigated because not all hospitals have access to ERCP, interventional radiology and hepatobiliary surgery at night or on week-ends. Transferring critically ill patients with AC could be a possibility, but remains challenging in case of uncontrolled acute circulatory failure [11–14]. In our observational “real life” study, we collected data from different regions in France including centers that may or may not have access to ERCP/interventional radiology. AC-associated mortality was not different between centers and we found that delayed biliary drainage (after 48 h) is associated with in-hospital mortality, after adjustment on several confounders including disease severity and appropriate empirical antimicrobial therapy. Previous studies on hospitalized AC patients have reported that delayed time to decompression was associated with longer lengths of hospital stay (> 24 h) [15], prolonged duration of organ failure (> 48 h) [14] or increased hospital readmission (> 48 h) [16]. However, none of them addressed the question of when to perform biliary decompression in high-risk AC patients with organ failure. Karvellas et al. found that delaying biliary decompression after 12 h was associated with increased mortality in patients with septic shock, whereas we identified a cut-off at 48 h. Such discrepancy could be explained by several differences in terms of population, drainage procedure or global mortality. This result highlights that the absence of ERCP availability during the entire week-end is a major issue, especially for patients admitted in ICUs on Fridays. Opening interventional endoscopic centers on Saturdays or Sundays could be an interesting option to improve outcome of critically ill patients with AC admitted in ICUs at the end of the week. Our results also highlighted that delayed biliary decompression > 24 h after ICU admission was not associated with higher mortality, suggesting that the first hours of AC patients management should mainly be devoted to resuscitation including antibiotic therapy and organ support therapy (mechanical ventilation, vasopressors, fluids) but not on emergency biliary decompression, especially if the patient has to be transferred to another hospital for ERCP. It is only after hemodynamic optimization, that biliary decompression should be considered. Such a conclusion based on retrospective data has to be confirmed prospectively.

In our study, patients were managed according to the International Guidelines with early resuscitation, biliary drainage (87%) and appropriate empirical antibiotic therapy (75%). Using multivariate analysis, we found that appropriate empirical antibiotic therapy is a protective factor, corroborating with Karvellas and colleagues’ conclusion as well as with studies focusing on peritonitis [17], nosocomial pneumonia [18] and acute bacterial meningitis [19]. First line antibiotic therapy must target gram negative bacteria, mainly *Enterobacteriaceae* that are frequently found both in blood and bile cultures. Interestingly, identifying ESBL-producing bacteria is not rare. Their prevalence seems to increase over time, suggesting that combining aminoglycoside with a β-lactam drug to broaden the spectrum should be recommended in critically ill patients with AC, especially with those having non-gallstone obstacles.

Patients had severe ACs with high SOFA scores and frequent use of organ support therapies. In-hospital mortality was 29% and decreased overtime. Mortality is lower than that reported in Karvellas*’study* [11]. However, these authors exclusively included patients with AC and shock whereas in our study, only 61% of the patients received vasopressors. In addition, strategy for biliary decompression was different, as in their study percutaneous biliary drainage was used as the first-line technique for sepsis source control whereas in our cohort, ERCP was performed in 76% of the cases. We found that ERCP performed in critically ill patients is safe with low complication rates, most of them being mild bleeding. In addition, ERCP was efficient, considered as optimal in 70% of the case. Non-optimal procedure may be due to infiltrating tumor-related AC supporting that a non-gallstone biliary obstacle is an independent pejorative prognosis factor [13, 20].

Most descriptive studies on AC evaluated hospitalized patients [21–23], and few data is available on critically ill patients admitted in ICUs. Comorbidities and pancreato-biliary malignancies were more frequent in our study, in comparison with Gomi and colleague’s study, which included patients from gastroenterology departments [24]. One important take-home message for clinicians is that fever being less frequently observed in our cohort, diagnosis of AC should not be ruled out in the absence of fever. We speculated that low frequency of fever in our cohort could be explained, at least in part, by antibiotic administration before ICU transfer as well as antipyretic use. In our cohort, blood cultures were positive in 68% of the cases, which is similar to the proportion previously reported on patients with AC and shock. However, it is twofold higher than in Lee and colleague’s study [21] which included all hospitalized patients with AC. In the latter study, bacteremia was associated with persistent organ failure at Day 2 [14] whereas in ICUs, positive blood culture was not identified as an independent predictive factor of mortality [25].

Our study should be interpreted in the light of its strengths and limitations. We have included patients from 11 ICUs across several geographic regions in France over a 14-year period, making this study the largest to date on patients with acute cholangitis managed in ICUs. The observed results are thus widely generalizable. Regarding its limitations, this study being an observational retrospective study, only association and not causality can be inferred. Given that this study was observational, we cannot conclusively exclude sources of selection bias. Finally, some data are lacking such as the time between symptom onset and ICU admission and also the causes of delayed biliary decompression.

## Conclusion

In this large multicenter study, we showed that acute cholangitis is a severe condition leading to high mortality in ICU patients. Severity of organ failure, causes of obstruction and local complications of AC are risk factors for mortality, as well as delayed biliary drainage. The impact of biliary decompression timing on mortality has to be prospectively evaluated in future studies.

## Supplementary Information

**Additional file 1.** Flow chart. **Additional file 2**. Number of patients with AC included in each center. **Additional file 3**. Variables associated with EBSL infection. **Additional file 4**. AC-associated mortality in participating centers. Crude mortality rate by center (left) and distribution of center effects on mortality rate (right) adjusted on individual confounders. Centers are sorted by study size. Black squares represent adjusted center effects on the mean mortality risk as odds ratio (OR) (comparison of each center to a theoretical average reference center with OR = 1).

## Data Availability

Not applicable.

## Abbreviations

AC: Acute cholangitis
ERCP: Endoscopic Retrograde Cholangiopancreatography
ESBL: Extended spectrum beta lactamase
ICU: Intensive care unit
OR: Odds ratio
MAP: Mean arterial pressure
SOFA: Sequential organ failure assessment
SAPS II: Simplified acute physiologic score II
