# Supplementary material for: Acute cholangitis in intensive care units: clinical, biological, microbiological spectrum and risk factors for mortality: a multicenter study
Source: Crit Care. 2021 Feb 6;25:49. doi: 10.1186/s13054-021-03480-1 (PMC7866656; doi:10.1186/s13054-021-03480-1)
Supplement: Supplementary file 1 — Additional file 1. Flow chart. Additional file 2. Number of patients with AC included in each center. Additional file 3. Variables associated with EBSL infection. Additional file 4. AC-associated mortality in participating centers. Crude mortality rate by center (left) and distribution of center effects on mortality rate (right) adjusted on individual confounders. Centers are sorted by study size. Black squares represent adjusted center effects on the mean mortality risk as odds ratio (OR) (comparison of each center to a theoretical average reference center with OR = 1). [file 13054_2021_3480_MOESM1_ESM.pptx]

## Slide 1
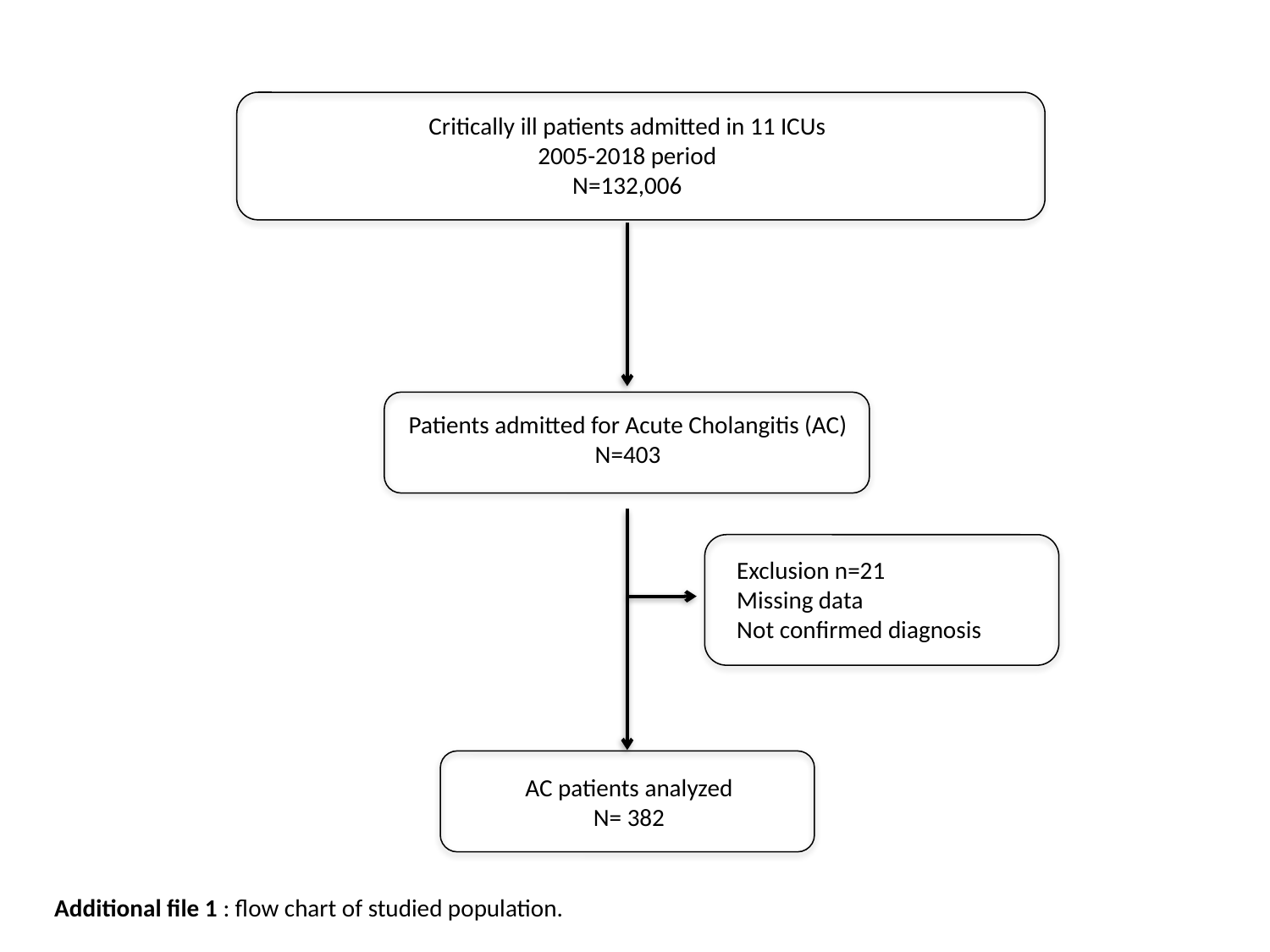

Critically ill patients admitted in 11 ICUs
2005-2018 period
N=132,006
Patients admitted for Acute Cholangitis (AC)
N=403
Exclusion n=21
Missing data
Not confirmed diagnosis
AC patients analyzed
N= 382
Additional file 1 : flow chart of studied population.

## Slide 2
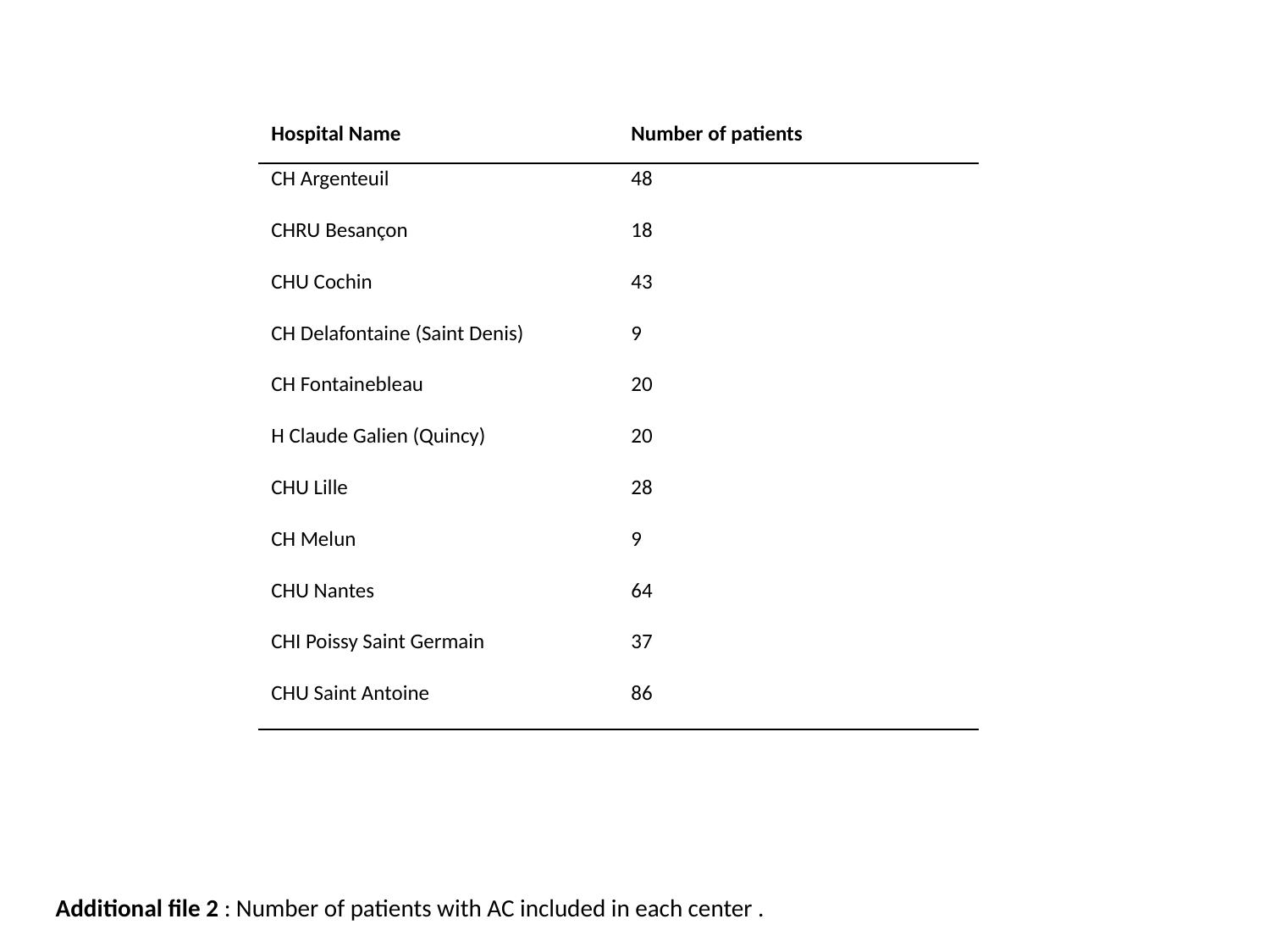

| Hospital Name | Number of patients |
| --- | --- |
| CH Argenteuil | 48 |
| CHRU Besançon | 18 |
| CHU Cochin | 43 |
| CH Delafontaine (Saint Denis) | 9 |
| CH Fontainebleau | 20 |
| H Claude Galien (Quincy) | 20 |
| CHU Lille | 28 |
| CH Melun | 9 |
| CHU Nantes | 64 |
| CHI Poissy Saint Germain | 37 |
| CHU Saint Antoine | 86 |
Additional file 2 : Number of patients with AC included in each center .

## Slide 3
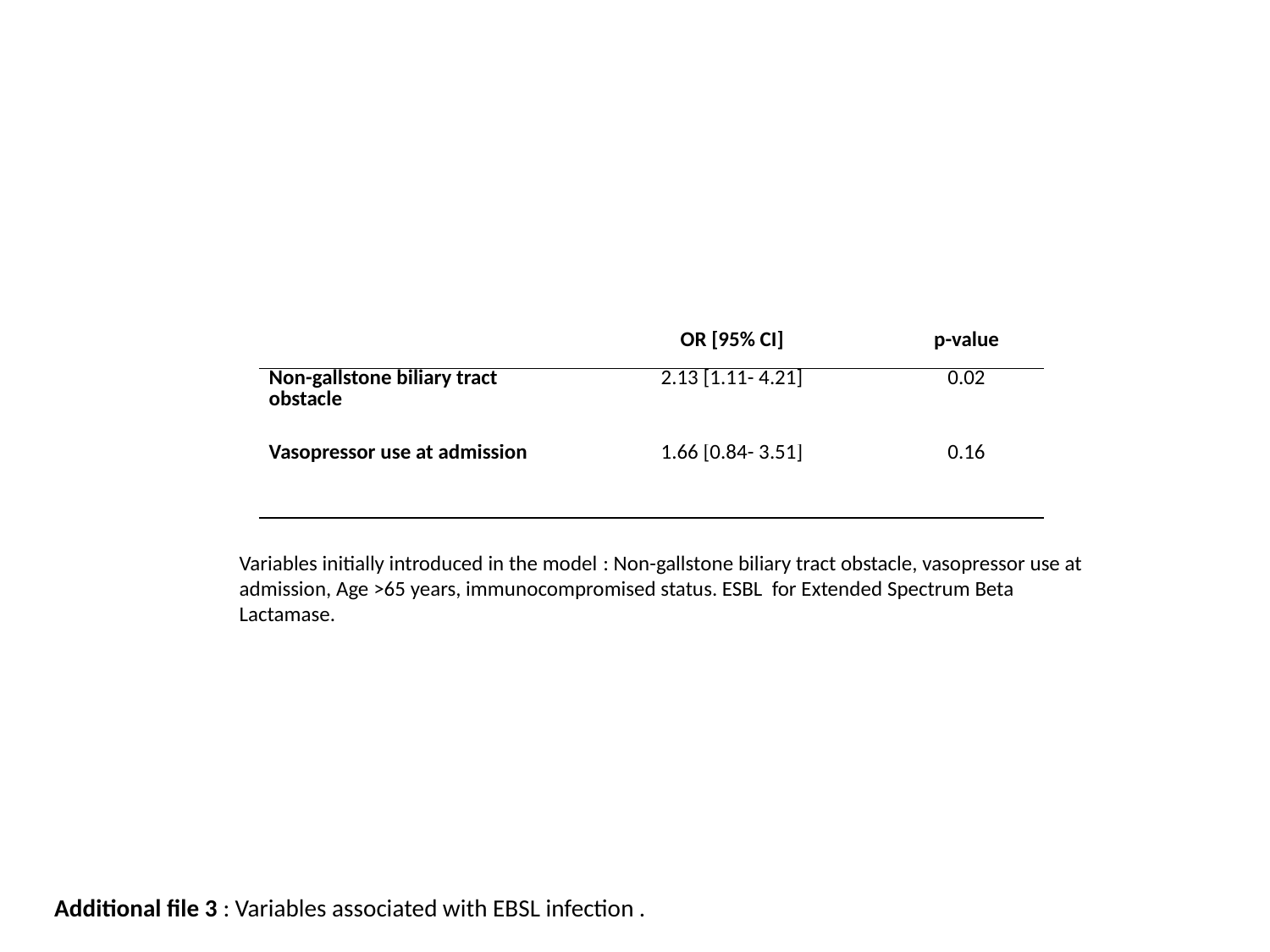

| | OR [95% CI] | p-value |
| --- | --- | --- |
| Non-gallstone biliary tract obstacle | 2.13 [1.11- 4.21] | 0.02 |
| Vasopressor use at admission | 1.66 [0.84- 3.51] | 0.16 |
Variables initially introduced in the model : Non-gallstone biliary tract obstacle, vasopressor use at admission, Age >65 years, immunocompromised status. ESBL for Extended Spectrum Beta Lactamase.
Additional file 3 : Variables associated with EBSL infection .

## Slide 4
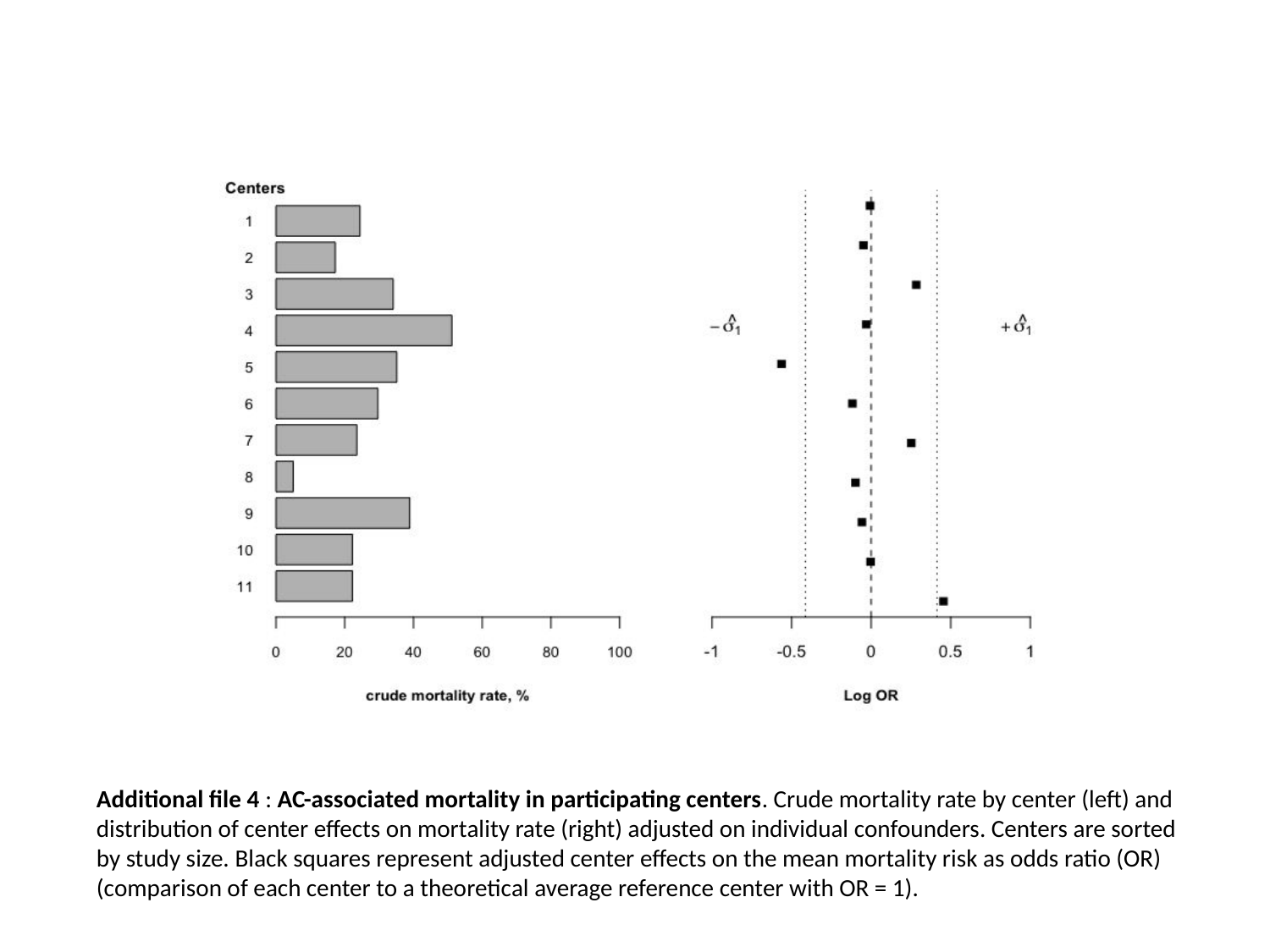

Additional file 4 : AC-associated mortality in participating centers. Crude mortality rate by center (left) and distribution of center effects on mortality rate (right) adjusted on individual confounders. Centers are sorted by study size. Black squares represent adjusted center effects on the mean mortality risk as odds ratio (OR) (comparison of each center to a theoretical average reference center with OR = 1).
